# PCSK9 is Expressed in Human Visceral Adipose Tissue and Regulated by Insulin and Cardiac Natriuretic Peptides

**DOI:** 10.3390/ijms20020245

**Published:** 2019-01-09

**Authors:** Marica Bordicchia, Francesco Spannella, Gianna Ferretti, Tiziana Bacchetti, Arianna Vignini, Chiara Di Pentima, Laura Mazzanti, Riccardo Sarzani

**Affiliations:** 1 Internal Medicine and Geriatrics, Department of Clinical and Molecular Sciences, University “Politecnica delle Marche”, 60126 Ancona, Italy; marica.bordicchia@gmail.com (M.B.); fspannella@gmail.com (F.S.); chiara.dipentima@live.it (C.D.P.); 2 Internal Medicine and Geriatrics, “Hypertension Excellence Centre” of the European Society of Hypertension, IRCCS-INRCA, 60127 Ancona, Italy; 3 Department of Clinical Sciences, Section of Biochemistry, Biology and Physics, School of Nutrition, University “Politecnica delle Marche”, 60126 Ancona, Italy; g.ferretti@univpm.it (G.F.); t.bacchetti@univpm.it (T.B.); a.vignini@univpm.it (A.V.); l.mazzanti@univpm.it (L.M.)

**Keywords:** PCSK9, natriuretic peptides, adipose tissue, lipid metabolism, LDL receptor, insulin

## Abstract

Proprotein convertase subtilisin/kexin type 9 (PCSK9) binds to and degrades the low-density lipoprotein receptor (LDLR), contributing to hypercholesterolemia. Adipose tissue plays a role in lipoprotein metabolism, but there are almost no data about PCSK9 and LDLR regulation in human adipocytes. We studied PCSK9 and LDLR regulation by insulin, atrial natriuretic peptide (ANP, a potent lipolytic agonist that antagonizes insulin), and LDL in visceral adipose tissue (VAT) and in human cultured adipocytes. *PCSK9* was expressed in VAT and its expression was positively correlated with body mass index (BMI). Both intracellular mature and secreted PCSK9 were abundant in cultured human adipocytes. Insulin induced PCSK9, LDLR, and sterol-regulatory element-binding protein-1c (SREBP-1c) and -2 expression (SREBP-2). ANP reduced insulin-induced PCSK9, especially in the context of a medium simulating hyperglycemia. Human LDL induced both mature and secreted PCSK9 and reduced LDLR. ANP indirectly blocked the LDLR degradation, reducing the positive effect of LDL on PCSK9. In conclusion, *PCSK9* is expressed in human adipocytes. When the expression of PCSK9 is induced, LDLR is reduced through the PCSK9-mediated degradation. On the contrary, when the induction of PCSK9 by insulin and LDL is partially blocked by ANP, the LDLR degradation is reduced. This suggests that NPs could be able to control LDLR levels, preventing PCSK9 overexpression.

## 1. Introduction

Maintenance of optimal blood lipid levels is central to vascular health. Liver plays a key role in lipoprotein metabolism, but much less is known about the role of adipose tissue. The adipose tissue is involved in energy balance and energy storage. It has endocrine functions and plays a fundamental role in the metabolism of triglyceride-rich lipoproteins. Recent studies suggest that thermogenic “brown” adipocytes are also involved in lipoprotein metabolism. Brown adipose tissue not only takes up triglycerides derived from plasma triglyceride-rich lipoproteins, but is also actively involved in the metabolic flux of high-density lipoprotein (HDL)-cholesterol to the liver [1].

Besides its role in triglyceride storage, adipose tissue contains a very large pool of free cholesterol, and adipocytes are known to support cholesterol efflux to HDL and apoA-I in vitro [2,3]. In fact, the main functional receptors for HDL, such as the ATP-binding cassette subfamily A member 1 (ABCA1) and the scavenger receptor class B type I (SR-BI), are expressed in mature adipocytes. Zhang et al. demonstrated that these receptors control cholesterol efflux. Furthermore, they suggested that adipose dysfunction caused by “inflammation”, as seen in the insulin-resistance conditions, may impair HDL lipidation in the adipocytes, reducing circulating HDL-C levels [4]. 

On the contrary, very little is known about the adipocyte role in low-density lipoprotein (LDL) handling and about the involvement of proprotein convertase subtilisin kexin type 9 (PCSK9) on LDL receptor (LDLR) regulation. About forty years ago, some landmarking studies were carried on LDLR and adipocytes. In 1979, Angel et al. demonstrated that isolated human adipose cells contain a high-affinity receptor which can bind, internalize, and degrade LDL, suggesting that adipose tissue is an important site of LDL and HDL interactions [5]. From that time, the role of adipose tissue in lipoprotein metabolism has been largely forgotten or not widely studied.

PCSK9, a member of the proprotein convertase family, behaves mainly as a chaperon and it is highly expressed in human liver [6]. A PCSK9 gain-of-function mutation was identified as a cause of autosomal dominant familial hypercholesterolemia [7]. Indeed, PCSK9 plays a critical role in the regulation of cholesterol homeostasis. Many studies have demonstrated its involvement in the regulation of LDL cholesterol (LDL-C) levels by controlling the recyclable LDLR on hepatocyte [8,9]. When PCSK9 is released from the Golgi apparatus into the circulation, it is able to induce the degradation of LDLR after its binding with LDLR-LDL-C hepatocyte surface complex [7,10,11]. The LDLR, the hydroxy-methyl-glutaryl CoA (HMG-CoA) reductase, and the PCSK9 are co-regulated by the sterol regulatory element binding protein-2 (SREBP-2), to prevent excessive cholesterol uptake and preserve cholesterol homeostasis [12]. Therefore, pharmacological activation of the *SREBP* pathway by HMG-CoA reductase inhibitors (statins) induce *PCSK9* expression in experimental and clinical settings [13,14]. Furthermore, *SREBP-1c* also appeared to be involved in the induction of *PCSK9* by insulin [12,15,16]. The role of SREBP-1c in the regulation of PCSK9 levels has also been observed in humans, where PCSK9 is positively correlated with insulin resistance, liver steatosis, and very low-density lipoprotein-triglyceride (VLDL-TG) levels [17]. This evidence suggests that PCSK9 may be also implicated in the metabolism of TG-rich lipoproteins, such as VLDL and intermediate-density lipoprotein (IDL), that can be uptaken by the LDLR via apoB and apoE binding.

Taken together, we suggest a role of adipose tissue in PCSK9-mediated lipoprotein metabolism, although there are almost no data about PCSK9 in human adipocytes. Recently, *PCSK9* was detected in mice perigonadal fat [18,19]. Mice lacking *PCSK9 (PCSK9−/−)* exhibit normal weight but increased visceral adipose tissue (VAT) due to adipocyte hypertrophy, independently from the LDLR. *PCSK9−/−* mice increased both fatty acid uptake and triglyceride synthesis in the adipose tissue, together with an increased surface density of VLDL receptor [18,19]. Insulin resistance, a condition found in PCSK9−/− mice, is linked with an impaired expression of natriuretic peptides (NPs) receptors in human VAT. Moreover, opposite effects of insulin and NPs have been documented regarding lipid storage in adipocytes, with NPs antagonizing insulin-stimulating TG storage [20,21].

Cardiac NPs, including type-A (ANP) and type-B (BNP), play a crucial role in maintaining cardiovascular homeostasis, given their impact not only on blood pressure regulation, but also on glucose and lipid metabolism [22,23]. They exert several actions on adipocytes through the activation of cGMP-dependent pathway, including activation of lipolysis [24,25] and lipid oxidation [26], together with thermogenic program [27]. An important inverse association has been found between plasma LDL-C and circulating NPs in subjects with a wide range of NT-proBNP levels [28]. 

In our study, the first step was to verify *PCSK9* expression and secretion in human VAT and human adipocytes. Once confirmed, we studied the reciprocal role of insulin and ANP in the regulation of *PCSK9* and *LDLR* expression, as well as their respective regulatory genes in human adipocyte cell model. We hypothesized that NP activity might influence adipose tissue lipoprotein metabolism, by regulating those proteins (PCSK9 and LDLR) that play a significant role in atherogenic dyslipidemia. 

## 2. Results

### 2.1. PCSK9 Expression in Human VAT

**Figure 1:**
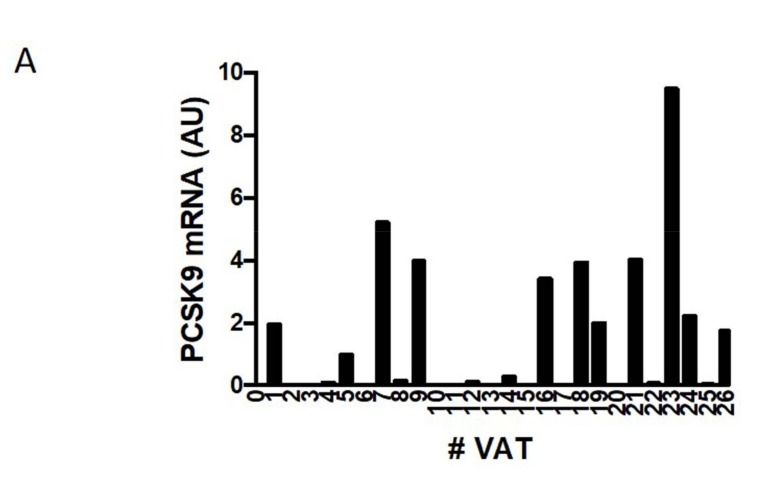

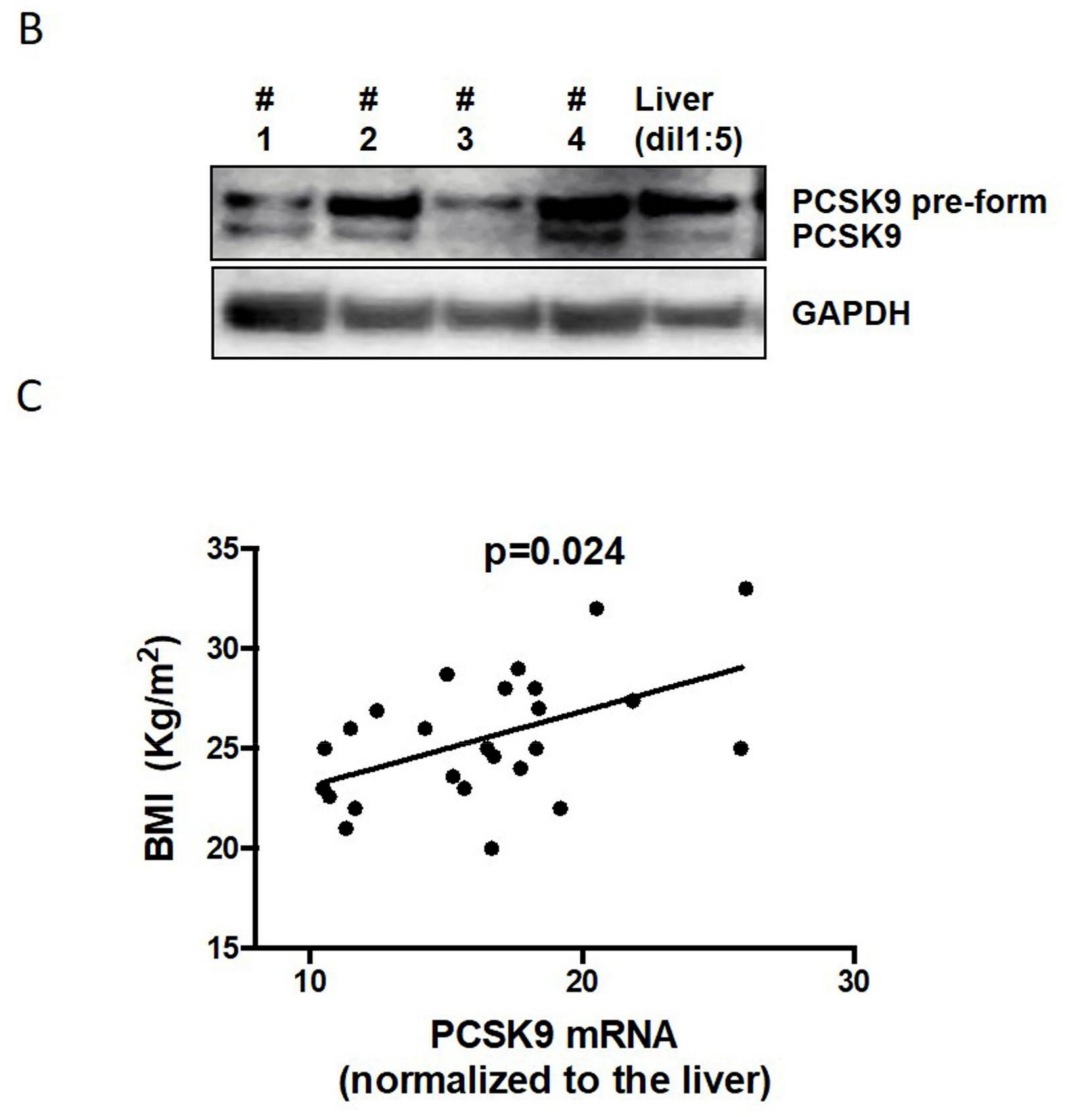
(**A**) *PCSK9* gene expression in VAT. Cycle threshold of *PCSK9* during real-time gene expression analysis for adipose tissue were between 21 and 24. (**B**) *PCSK9* levels in differentiated human adipocytes. (**C**) *PCSK9* in VAT according to BMI. # number of sample.

General characteristics of the studied population are summarized in Table 1. It is known that *PCSK9* is abundantly expressed in liver, small intestine, and kidney. Our present data show that *PCSK9* is abundantly expressed in human adipose tissue as well. Figure 1A shows *PCSK9* gene expression in VAT, even if highly variable among patients. The protein analysis of PCSK9 in human adipose tissue and liver revealed that the pre-form of *PCSK9* is easily detectable (72 kDa; Figure 1B). In fact, human PCSK9 is synthesized as a precursor that undergoes autocatalytic cleavage of its N-terminal prosegment in the endoplasmic reticulum (ER) necessary for its activation and function [6]. The mature form (63 kDa) is also detectable, but it is clearly weaker than pre-form, as expected. As shown in Figure 1C, *PCSK9* expression levels are significantly and positively correlated with the body mass index (BMI) of the 26 patients studied (*p* = 0.024), even after adjustment for gender and age (β = 0.429; 95% CI 0.023–1.224; *p* = 0.016). No significant correlation emerges between *PCSK9* in VAT and LDL cholesterol, according to linear regression model adjusted for gender and age (*p* = 0.654).

### 2.2. PCSK9 and LDLR Regulation in Human Adipocytes by Insulin

**Figure 2:**
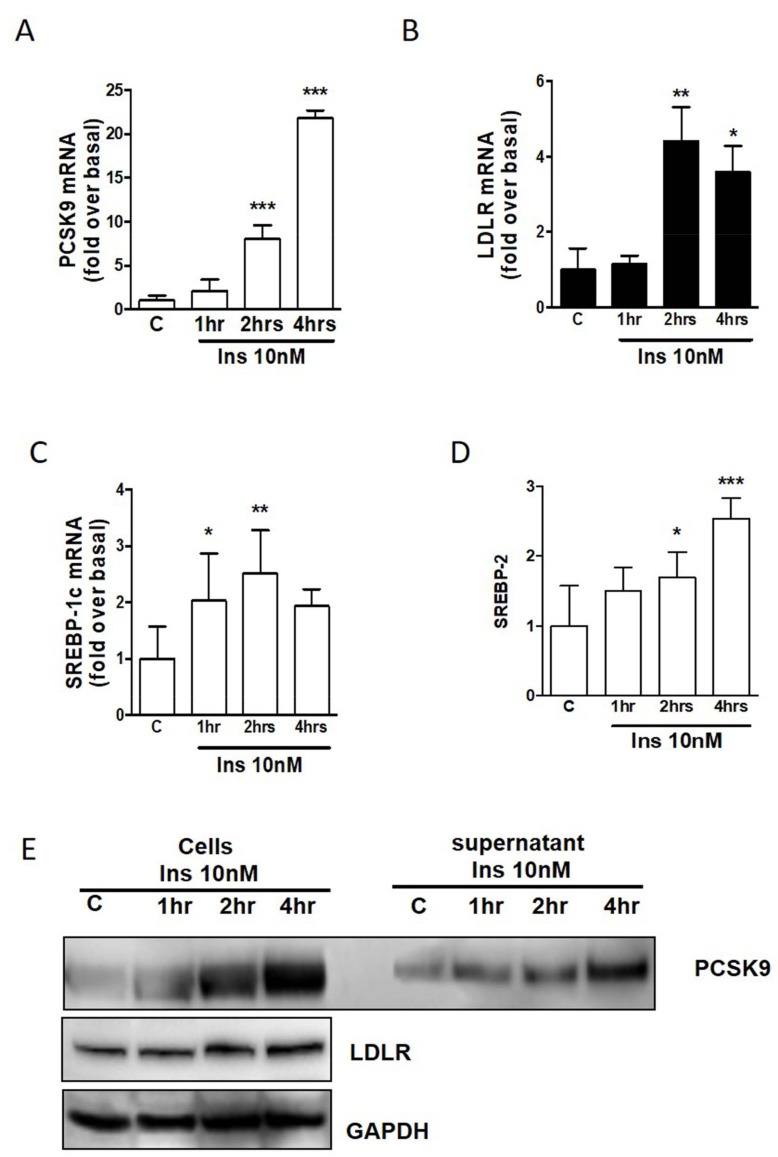
(**A**) *PCSK9* induction by insulin in differentiated human adipocytes. (**B**) *LDLR* induction by insulin in differentiated human adipocytes. (**C**) *SREBP-1c* induction by insulin in differentiated human adipocytes. (**D**) *SREBP-2* induction by insulin in differentiated human adipocytes. (**E**) PCSK9, LDLR, and GAPDH levels after treatment with insulin. * *p* < 0.05, ** *p* < 0.01 and *** *p* < 0.001 vs. control.

Differentiated adipocytes in multiple wells were treated for 1, 2, or 4 h with 10 nM of insulin. As shown in Figure 2A, *PCSK9* gene expression is 20-fold increased by insulin after 4 h, but a significant increase is already seen after 2 h. Similarly, *LDLR* (Figure 2B), *SREBP-1c* (Figure 2C), and *SREBP-2* (Figure 2D) are significantly increased by insulin with an earlier time course for the regulatory protein *SREBP-1c*. Protein analysis of PCSK9 confirms the induction of PCSK9 after insulin treatment. Interestingly, we analyzed the cell lysate as well as supernatant, and we observed that PCSK9 after 4 h treatment is increased, especially in the mature form secreted by adipocytes into the media (Figure 2E). LDLR protein is also induced by insulin in human adipocytes (Figure 2E).

### 2.3. PCSK9/LDLR Modulation by Cardiac Natriuretic Peptides

**Figure 3:**
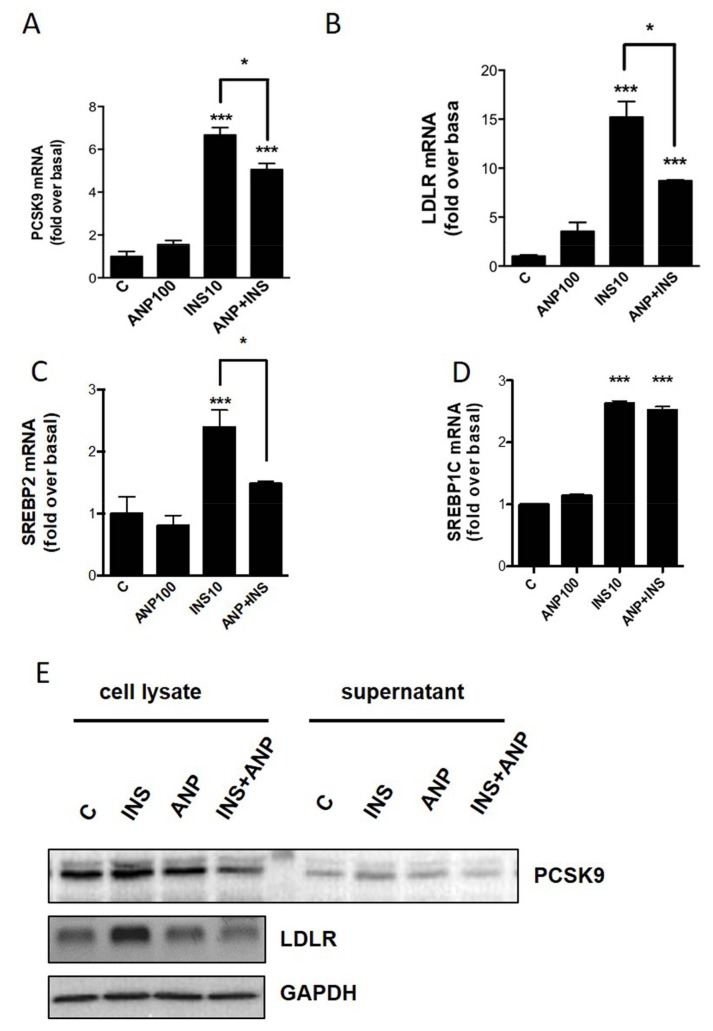
One-way ANOVA. (**A**) Effects of insulin and ANP on *PCSK9* in differentiated human adipocytes. (**B**) Effects of insulin and ANP on *LDLR* in differentiated human adipocytes. (**C**) Effects of insulin and ANP on *SREBP-2* in differentiated human adipocytes. (**D**) Effects of insulin and ANP on *SREBP-1c* in differentiated human adipocytes. (**E**) PCSK9, LDLR, and GAPDH levels after treatment with insulin and ANP. *** *p* < 0.001 vs control; * *p* < 0.05 insulin 10nM vs insulin+ANP treatment.

To understand whether PCSK9 and LDLR respond not only to insulin but also to NPs, that are physiologic antagonists of insulin effects on lipid metabolism in adipose tissue, adipocytes were treated for 4 h with insulin (10 nM), or ANP (100 nM), or insulin together with ANP. Gene expression analysis shows that *PCSK9* and *LDLR* are significantly induced by insulin, as described above, but also that ANP is able to partially block the insulin effect (Figure 3A,B). Considering the main genes involved in the regulation of cholesterologenesis, we found that *SREBP-2* is induced by insulin and ANP is able to block the insulin effect (Figure 3C). On the contrary, ANP is not able to reduce the induction of *SREBP-1c* by insulin (Figure 3D). Similar results for PCSK9 and LDLR were obtained using Western blot analysis (Figure 3E).

### 2.4. The Influence of LDL from Human Plasma

**Figure 4:**
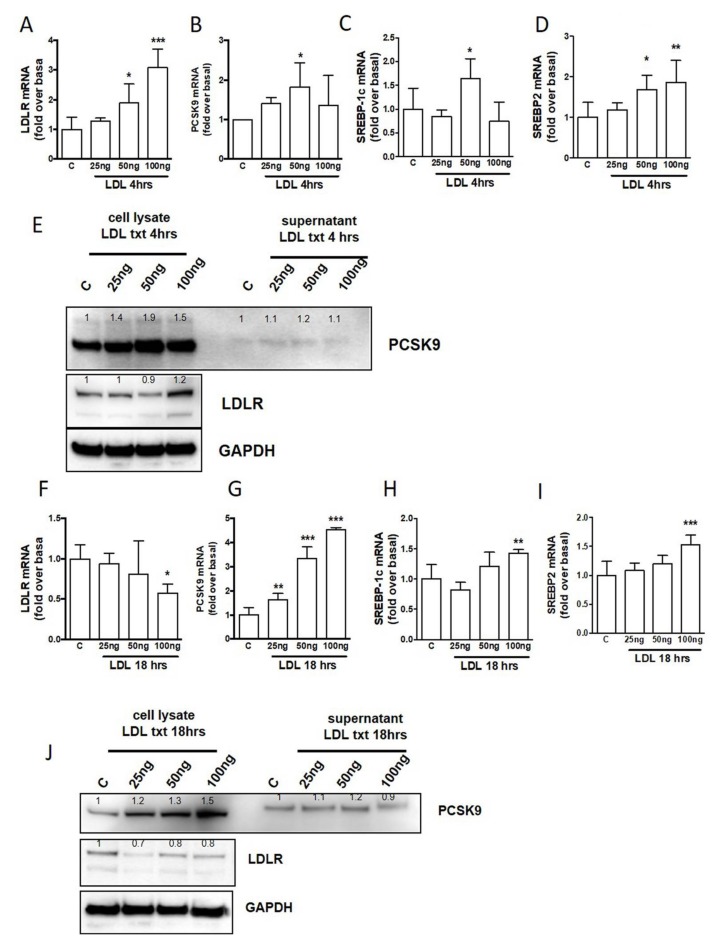
One-way ANOVA. (**A**) Effects of LDL treatment for 4 h on *LDLR* in differentiated human adipocytes. (**B**) Effects of LDL treatment for 4 h on *PCSK9* in differentiated human adipocytes. (**C**) Effects of LDL treatment for 4 h on *SREBP-1c* in differentiated human adipocytes. (**D**) Effects of LDL treatment for 4 h on *SREBP-2* in differentiated human adipocytes. (**E**) PCSK9, LDLR, and GAPDH proteins levels after LDL treatment for 4 h. (**F**) Effects of LDL treatment for 18 h on *LDLR* in differentiated human adipocytes. (**G**) Effects of LDL treatment for 18 h on *PCSK9* in differentiated human adipocytes. (**H**) Effects of LDL treatment for 18 h on *SREBP-1c* in differentiated human adipocytes. (**I**) Effects of LDL treatment for 18 h on SREBP-2 in differentiated human adipocytes. (**J**) PCSK9, LDLR, and GAPDH proteins levels after LDL treatment for 18 h. * *p* < 0.05, ** *p* < 0.01 and *** *p* < 0.001 vs control.

To study the physiological mechanism that links adipocyte PCSK9 with circulating LDL, human adipocytes were treated with increasing concentrations of isolated LDL from human plasma (from 25 to 100 ng/mL) for 4 and 18 h. Gene expression analysis shows that at the first time point (4 h, Figure 4A–D) the treatment with LDL induces a significant increase of all the target genes: *PCSK9, LDLR, SREBP-1c*, and *SREBP-2*. Interestingly, at the second time point (18 h) it is evident that human LDL decreases *LDLR* but, at the same time, significantly increases *PCSK9* together with *SREBP-1c* and *SREBP-2* (Figure 4F–I). Protein analysis clearly shows that, during the incubation times, PCSK9 initially increases as a mature form into the cells (Figure 4E) and then, after 18 h, increases as the mature form secreted into the media (Figure 4J). At the same time, LDLR increases after 4 h treatment (Figure 4E) and, thereafter, is significantly reduced after 18 h (Figure 4J). These time-dependent responses suggest that, at the beginning, the presence of LDL induces all the physiological pathways involved in cholesterol uptake, but after 18 h, the strong induction of PCSK9, especially for the secreted mature form, may induce LDLR reduced function by degradation.

### 2.5. LDL and ANP Effects on Human Adipocytes

**Figure 5:**
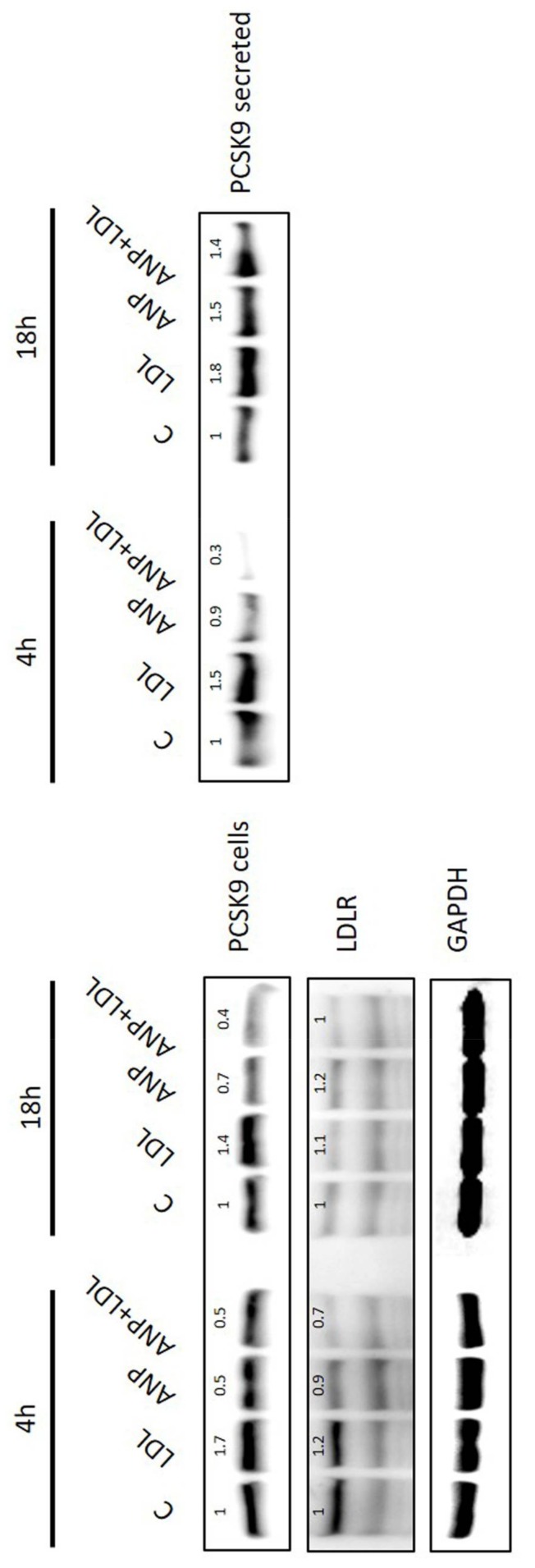
PCSK9, LDLR, and GAPDH levels after treatment with LDL and ANP for 4 and 18 h.

*PCSK9* and *LDLR* expression were also analyzed in presence of isolated LDL (50 ng/mL) and ANP (100 nM). As shown in Figure 5, protein analysis reveals that 4 h of treatment with LDL significantly induces PCSK9, as well as LDLR, and that this effect is blocked by ANP. After 18 h, it is still evident that, while PCSK9 is clearly induced also in the secreted form, LDLR is reduced, as described in Figure 4. Interestingly, ANP is still able to block the effect of LDL on target genes after 4 and 18 h treatments (Figure 5).

## 3. Discussion

The focus of this study was to investigate the expression and regulation of PCSK9 in human adipose tissue and adipocytes, using insulin and ANP, two opposing hormones on lipid metabolism [20]. It is known that the active form of PCSK9 is derived from three different steps: the cleavage of the signal sequences, the intramolecular proteolysis of proPCSK9, and trafficking through the *trans*-Golgi network before the secretion into extracellular space, where it reaches its target, LDLR [6,29,30]. Gene expression and protein analysis revealed that the precursor form of *PCSK9* is expressed and easily detectable in human visceral perirenal fat, even if there are wide differences among subjects. The concentrations of *PCSK9* in adipose tissue positively correlated with BMI values, the most used clinical index of adiposity. Interestingly, PCSK9 plasma concentrations were also related to carotid artery intima-media thickness (cIMT) in overweight and obese individuals, in comparison with the normal weight group, suggesting that PCSK9 could be an indicator as well as a player of cardiometabolic and vascular changes induced by excessive adiposity [31].

Therefore, we investigated the regulation of PCSK9 using the Simpson–Golabi–Behmel syndrome (SGBS) human adipocyte cell line. Differentiated SGBS adipocytes were used to verify the ability of insulin, ANP, and LDL to regulate PCSK9. First, we showed that 10 nm of insulin strongly induced PCSK9 and LDLR after 2 and 4 h of treatment. It has been reported that LDLR and PCSK9 share SREBP-2 as a common regulatory pathway, and that *PCSK9* expression is also regulated by insulin via the *SREBP-1c* in primary mouse and rat hepatocytes, as well as in vivo, during hyperinsulinemic-euglycemic clamps. [32]. These *SREBPs* seem to be active also in adipocytes. Indeed, *SREBP-1c* is activated by insulin earlier than *SREBP-2*, being already significantly increased after 1 h. This suggests that *SREBP-1c* is involved in *PCSK9* regulation in human adipocytes.

We recently described that higher insulin levels, together with higher glucose concentration, simulate insulin resistance found in obese patients, and reduced the ability of NPs to induce lipolysis and the thermogenic pathway [20]. Some interactions between the PCSK family and NPs have been recently discovered, such as for PCSK6, that activates the corin, a key enzyme in the activation of ANP precursors [33,34]. Here, we show that ANP is able to reduce the insulin-mediated induction of PCSK9 and LDLR in human adipocytes. Our data also show that ANP is able to reduce the regulation of SREBP-2, but not of SREBP-1c. SREBP-1c preferentially activates genes involved in fatty acid biosynthesis or carbohydrate metabolism, including fatty acid synthetase, acetyl-CoA carboxylase, or glucokinase (GK) [32]. GK converts glucose into glucose 6-phosphate and, therefore, SREBP-1c is thought to have a permissive action on glucose-dependent gene regulation [35]. Moreover, *SREBP-1c* is induced by *LXRalpha*, that is stimulated by insulin independently by glucose concentration, as we previously described [20]. In the liver, SREBP-2 processing is also enhanced by peroxisome proliferator-activated receptor gamma (PPAR-gamma) activation, affecting both PCSK9 and LDLR expression [36]. It is known that PPAR-gamma has an important role in both cell differentiation and energy metabolism in adipocytes [37,38]. The role of PPAR-gamma activation on these proteins in human adipocytes could be an interesting aspect to be explored in future studies.

Given the close relation between circulating PCSK9 and LDL levels in human plasma [39,40,41], we tested the role of different concentrations of LDL on PCSK9 and LDLR expression in our human adipocyte cell model. We used LDL isolated from human plasma to test the functionality of the LDLR/PCSK9 system, and our data indicate a fully functional system. The object of the present study was only to evaluate the expression and regulation of PCSK9 mediated by insulin and ANP. Future research, focused on the direct role of PCSK9 and LDLR in VAT and the relationship between PCSK9 and the morphology of adipocytes (i.e., lipid droplet formation or differentiation status), is warranted.

Published data reported that expression of PCSK9 in cultured cells has variable effects on LDLR. In some cell types, such as human hepatoma cells (HepG2 and HuH7) or human embryonic kidney cells (HEK-293 cells), PCSK9 expression dramatically reduces LDLR levels [29,42,43]. In other cells types, including fibroblasts, Chinese hamster ovarian (CHO-K1), monkey kidney cells (COS7) and rat liver cells (McArdle RH7777), PCSK9 is not likely to affect LDLR expression [42,43,44]. Here, we found that human LDL, added to the cells culture media, initially—after 4 h treatment—induces LDLR and pre-form PCSK9. After 18 h of treatment, the high levels of mature PCSK9 are secreted into the media, and are able to induce the degradation of LDLR. In fact, after 18 h, LDLR protein levels are reduced compared with LDLR after 4 h LDL treatment.

Moreover, when we tested the effect of LDL combined with ANP, we observed that LDL induced LDLR and the non-secreted form of PCSK9 after 4 h. At the same time, ANP is able to partially block the LDL-induced regulation. After 18 h, we confirmed that the LDL-mediated induction of PCSK9 was still present, and the PCSK9 secreted form was also significantly induced.

The elevated secretion of PCSK9 could represent the mechanism for the reduction of LDLR. Indeed, in the cells treated with ANP, where PCSK9 is reduced, the levels of LDLR are stable, suggesting again that ANP, by blocking the induction of PCSK9, indirectly reduced the degradation of LDLR. 

**Figure 6:**
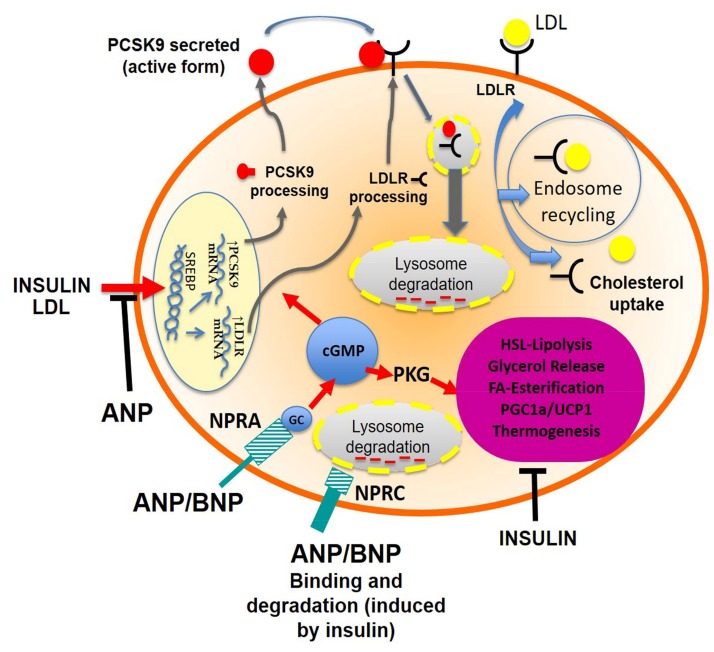
Interactions between insulin and cardiac natriuretic peptides (NPs) in lipid metabolism and PCSK9-LDL receptor handling. The potent lipolytic activity of NPs (ANP and BNP secreted from the heart), mediated by NPRA, is strongly reduced by insulin through the induction of the clearance receptor (NPRC), which binds and degrades NPs. Insulin also induces PCSK9, but this effect is opposed by NPs, especially in conditions simulating hyperglycemia. Overall, the NPs are likely to reduce the triglycerides and to increase cholesterol in human adipocytes, opposing insulin effects. Such activities may also have systemic consequences on blood lipid levels. T-bar arrow indicates inhibitory activity.

Although in our research the regulatory effect of ANP appears to be modest, to the best of our knowledge, this is the first study demonstrating that *PCSK9* is expressed in adipose tissue, and ANP is able to interact with PCSK9 pathways. Several studies showed the role of NPs on lipid metabolism and adipose tissue, but the interaction of ANP with cholesterol metabolism through PCSK9 and LDLR regulation is absolutely new. ANP partially blocked the effect of insulin and LDL in adipocytes, but it was more effective in culture conditions simulating hyperglycemia. Overall, NPs are likely to reduce triglycerides and to increase cholesterol in human adipocytes, opposing insulin effects. Such activities may also have systemic consequences on blood lipid levels (Figure 6). We believe that this study could be the first of future studies on human adipose tissue, in order to better explain which mechanisms are involved in lipid metabolism.

In conclusion, our data show a potentially relevant role for adipose tissue and adipocytes in the regulation of PCSK9-LDLR in humans, similar to what is known for human liver. NPs appear to play a key role in this pathway, with potentially important implications, especially in patients with obesity and hypertension, in which NPs could be able to regulate not only the blood pressure, but also the LDLR levels, preventing PCSK9 overexpression.

## 4. Materials and Methods

### 4.1. Reagents and Antibodies

Insulin, dexamethasone, isobutylmethylxanthine, tri-iodothyronine, transferrin, and wortmannin were obtained from Sigma-Aldrich (St. Louis, Missouri, USA). Antisera against PCSK9 and LDLR were from Abcam (Abcam, Cambridge, MA, USA). GAPDH antibody (cat #SC25778) and secondary antibody anti-rabbit (cat #SC2054) were from Santa Cruz Biotech; CA, USA. SuperSignal West Femto Maximum Sensitivity Substrate was from Thermo Scientific, Rockford, IL, USA.

### 4.2. Human Adipose Tissue

A set of human VAT samples (*n* = 26) were obtained from patients undergoing radical nephrectomy for localized clear cell renal carcinoma (without any evidence of local or metastatic cancer spread: T1/T2, N0, M0) at the “Ospedali Riuniti” University Hospital of Ancona, Italy. All women were in menopause. Patients with diabetes were excluded from the study, therefore, no patients took insulin or any other medications. The study was conducted in accordance with the guidelines proposed in The Declaration of Helsinki and the local Ethics Committee approved the study protocol (ID: 206964, date: 28 September 2006). All patients gave written informed consent for the collection of clinical data and tissue samples.

### 4.3. Adipocyte Cells Culture

Cells from Simpson–Golabi–Behmel syndrome cell line (SGBS) were grown in growth medium (DMEM/F12 with 10% fetal calf serum). When adipocytes reached 85%–90% confluence, they were differentiated in differentiation medium that included insulin, as previously described. To test the acute effect of insulin on NP receptors, we removed insulin from differentiation media at day 7 instead of day 11/12, as previously described [20]. Thus, on the seventh day, the cells were washed and deprived of insulin at least for three days, and were then treated with insulin and/or atrial natriuretic peptide (ANP). To assess whether PCSK9 expression and related genes are modulated by insulin, differentiated adipocytes were treated with 10 nM of insulin for 1, 2, and 4 h. PCSK9 regulation was also evaluated in response to cardiac NPs. Adipocytes were treated with 100 nM of ANP, as used in previous work [20]. ANP was also used together with insulin to evaluate the relative power of these two physiological counteracting (lipolytic vs. lipogenic) hormones on PCSK9/LDLR. At least 6 different wells in 3 different experiments were performed for each treatment.

### 4.4. Isolation of Human Plasma LDL

The role of LDL on PCK9 and LDLR expression was studied using LDL separated from human plasma. Fresh pooled human plasma from fasting healthy young volunteers (coauthors of these manuscript) was used for the preparation of LDL. LDL (density between 1.025 and 1.063 g/mL) were isolated by single vertical spin gradient ultracentrifugation, as described by Chung et al. [45]. After dialysis at 4 °C for 24 h against 10 mmol/L PBS (pH 7.4), LDLs were concentrated in a SpeedVac Concentrator and LDL protein concentration was determined by the method of Bradford [46].

The first set of experiments was performed to verify the effect of different concentrations of LDLs, from 25 to 100 ng/mL, after 4 and 18 h of incubations. Subsequently, PCSK9 and LDLR were analyzed after 4 and 18 h of treatment with human LDL (50 ng/mL), ANP, or together (ANP + LDL).

### 4.5. RNA Isolation and Gene Expression Analysis

Total RNA was extracted using TRIzol (Invitrogen, Carlsbad, CA, USA) and RNA reverse transcription of 2 μg was performed with High-Capacity cDNA Reverse Transcription Kit with RNase Inhibitor (Applied Biosystems, Warrington, UK). All gene expression experiments in SGBS and primary VAT adipocytes cultures were analyzed with SYBR Select Master Mix (Applied Biosystems Darmstadt, Germany). Each single gene expression experiment was performed in triplicate. Differences in total RNA or different efficiency of cDNA synthesis among samples were normalized using human GAPDH expression.

### 4.6. Western Blotting

Treated cells were lysed and sonicated in an appropriate buffer, as previously described [27]. Protein concentrations were determined using the Bradford Assay (Biorad, Hercules, CA, USA) and 50 μg of total proteins was resolved in 12% sodium dodecyl sulfate-polyacrylamide gel electrophoresis (SDS-PAGE), transferred to a PVDF membrane (Immobilon P, Millipore, Burlington, MA, USA), and probed overnight at 4 °C with specific PCSK9 or LDLR primary antibodies. Secondary antisera against rabbit IgG conjugated with peroxidase was used for specific protein detection. Target proteins were visualized using an enhanced chemiluminescent substrate (SuperSignal West Femto Maximum Sensitivity Substrate, Pierce) and were measured in comparison with GAPDH (Santa Cruz Biotech, Dallas, TX, USA). Image acquisition was performed on an ALLIANCE MINI HD9 (UVITEC, Cambridge UK). All lines were quantified with UVI-TEC NineAlliance analysis software and each line sample of Western blot shows the relative number of quantification, compared with control, that was considered as 1 (100%). In some cases, membranes were ‘stripped’ by incubation in a buffer (0.76 g Tris, 2 g SDS, 700 μL β-mercaptoethanol in 100 mL) at 37 °C for 45 min, in order to be subsequently probed with additional antibodies.

### 4.7. Statistical Analysis

Results are presented as mean ± SEM, unless otherwise indicated. Data were analyzed using two-tailed Student’s *t*-test, one-way ANOVA, followed by post hoc Newman–Keuls tests when *F* was significant. A non-parametric test for two related samples (Wilcoxon’s signed ranks test) was used to identify differences between each treated group and controls, differences between more than 2 groups were analyzed by analysis of variance and *post hoc* Holm-Bonferroni test. Pearson’s correlation coefficient was used to assess the association between PCSK9 gene expression and BMI. Multiple linear regression was used to create adjusted models. SPSS 11.0 software was used for statistical analysis (SPSS Inc., Chicago, IL, USA) and a *p* < 0.05 was considered significant.

## Figures and Tables

**Table 1:** General characteristics of studied population.

Variables	*N*	Mean ± SE
Gender (male/female)	26	15/11
Age (y)	26	66.9 ± 1.4
BMI (kg/m^2^)	26	25.6 ± 0.8
Waist (cm)	26	96.9 ± 1.9
SBP (mmHg)	24	139.1 ± 3.1
DBP (mmHg)	24	78.9 ± 2.0
Triglycerides (mg/dL)	17	125.4 ± 9.7
Total Cholesterol (mg/dL)	17	176.1 ± 11.7
HDL Cholesterol (mg/dL)	17	39.5 ± 2.7
LDL Cholesterol (mg/dL)	17	118.2 ± 10.1
Non-HDL Cholesterol (mg/dL)	17	139.7 ± 9.9

BMI: body mass index; SBP: systolic blood pressure; DBP: diastolic blood pressure; HDL: high-density lipoprotein; LDL: low-density lipoprotein.
